# Control over the Morphology of Dried Supraparticles from the Evaporation-Induced Self-Assembly of Aerosol Droplets Containing Polydisperse Nanoparticles

**DOI:** 10.1021/acs.langmuir.6c02636

**Published:** 2026-08-29

**Authors:** Lukesh K. Mahato, Panagiotis G. Georgiou, Barnaby E. A. Miles, Sorrel K. Haughton, Jake G. Edmans, Rachael E. H. Miles, Steven P. Armes, Jonathan P. Reid

**Affiliations:** † School of Chemistry, 1980 University of Bristol, Bristol BS8 1TS, U.K.; ‡ School of Mathematical and Physical Sciences, Dainton Building, 5292 University of Sheffield, Sheffield S3 7HF, U.K.

## Abstract

Control over the fabrication and morphology of supraparticles by processes such as spray drying is critical for designing materials for specific applications. We demonstrate morphological control to prepare either spherical or buckled supraparticles via evaporation-induced self-assembly of aqueous aerosol droplets containing two or more populations of sterically-stabilized diblock copolymer nanoparticles of varying size. Supraparticles are prepared at a relative humidity of either 0% or 45% when using monomodal, bimodal, trimodal, tetramodal, or pentamodal nanoparticle dispersions. The final supraparticle morphology is controlled by adjusting the relative concentrations of nanoparticles of differing size to tune the mean nanoparticle diffusion coefficient, which is inversely proportional to the harmonic mean of the nanoparticle diameter. This harmonic mean-based approximation enables the final supraparticle morphology to be accurately predicted by controlling the diffusion coefficient and evaporation rate. This approach is valid for systems with moderate size disparities. However, if the smallest nanoparticles are more than ten times smaller than the largest nanoparticles, then diffusion-driven segregation of the smallest nanoparticles at the surface of the drying aqueous droplets determines the final supraparticle morphology.

## Graphical abstract

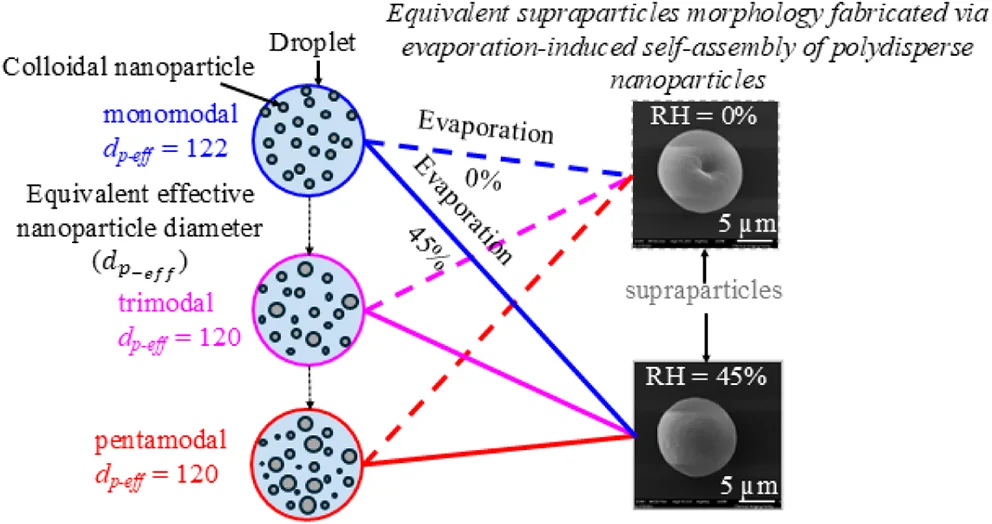

## 1 Introduction

A supraparticle is an assembly of primary colloidal particles such as silica, polystyrene, metal oxides or quantum dots.^1,2^ Supraparticles are of significant interest due to their tunable size, morphology and multifunctional characteristics.^3–7^ They may exhibit different physical and chemical properties compared to the corresponding primary nanoparticles owing to their internal morphology.^8^ For example, their larger size and reduced surface energy may lead to enhanced stability, collective optical or magnetic responses, and enhanced mechanical strength, making them highly advantageous for a broad range of applications.^9,10^ Control over the precise morphology of fabricated supraparticles is critical to optimize such materials for specific applications. For example, spherical supraparticles are advantageous for fluid transport, drug delivery system, aerosol formation, and the generation of ordered structures for photonic^11^ and catalytic applications due to their geometric uniformity, high stability (i.e., minimal surface area to volume ratio), and favorable flow dynamics (lower resistance to fluid flow).^12–14^ In contrast, hollow spherical supraparticles are ideal for encapsulation and for the design of low-density materials. In addition to the formation of porous or buckled morphologies that may exhibit higher surface-area-to-volume ratios for applications such as heterogeneous catalysis and adsorption,^15,16^ supraparticle engineering also plays an important role in the fabrication of dense, highly spherical microparticles. For example, spray drying can be used to produce compact ceramic or inorganic microspheres with enhanced mechanical properties and structural integrity. In this case, careful control over drying kinetics, solute concentration, and shell formation is critical to suppress buckling, avoid the formation of hollow spheres and achieve dense morphologies. More generally, these examples highlight that precise control over supraparticle morphology is important not only for tuning surface area and porosity, but also for designing mechanically robust and structurally uniform materials.^17,18^ Given the direct correlation between morphology and functionality, the reproducible fabrication of supraparticles with desirable morphologies under various conditions remains a pivotal challenge. One key question driving current research is how to precisely control final supraparticle morphology in processes such as evaporation-induced self-assembly by tailoring parameters such as the nanoparticle size distribution and the evaporation kinetics of the host droplets.

Various laboratory-scale methods have been developed for the fabrication and study of supraparticles, including spray-drying,^19^ levitated droplet evaporation,^20,21^ and sessile droplet evaporation.^22–24^ Among these, sessile droplet studies^25^ have been frequently employed for fundamental investigations of evaporation kinetics and supraparticle formation. However, such experiments have been typically limited to the formation of larger, millimeter-sized supraparticles and are not readily scalable to understand processes in smaller droplets or spray droplets. Moreover, while sessile droplets are relevant to systems where droplet-surface interactions strongly influence the final morphology, such as when the presence of a substrate can induce an uneven evaporative flux during drying, their relevance for free droplet drying is uncertain.^26–28^ Further, spray-drying enables higher production rates of supraparticles, but with less control over their size (∼0.1–600 μm).^10,29,30^

The addition of surfactants and solutes to control colloidal particle–particle interactions during drying and dried supraparticle morphology has been extensively studied.^31–33^ Surfactants can stabilize colloidal particles within the drying droplets by providing electrostatic or steric repulsion, thereby affecting the final supraparticle morphology.^34^ However, the efficacy of such stabilization is highly dependent on the nature of the colloidal particles. For example, sodium dodecyl sulfate (SDS) has been shown to destabilize certain spherical copolymer nanoparticles.^35,36^ Such aggregation highlights a critical limitation: the interaction between colloidal particles and surfactants is system-specific, governed by the chemical composition and surface charge of the surfactant, and the interfacial chemistry of the nanoparticles. As a result, surfactant-based approaches for controlled fabrication of supraparticles are not universally applicable. This underscores the need for alternative strategies for the precise and reproducible fabrication of supraparticles with desirable morphologies.

Recently, we demonstrated that well-defined supraparticles could be prepared by evaporation of aerosol droplets containing dispersions of monodisperse nanoparticles.^37,38^ Miles et al.^39^ reported that using larger nanoparticles reduces the degree of supraparticle buckling at a given relative humidity and temperature; buckled morphologies tend to be formed when using smaller nanoparticles owing to stronger capillary forces, which induce shell instability. Consistent with these measurements, the morphology of dried supraparticles can be predicted by determining the initial Peclet number (*Pe*),^19^ which is defined as the ratio of the initial evaporation rate of the droplet containing the suspension of nanoparticles to the diffusion coefficient of the nanoparticles within the drying droplet (see eq 1).

1 $$Pe=\frac{K}{8D}=\frac{-8R_{d}\frac{dR_{d}}{dt}}{8\left(\frac{k_{B}T}{3\pi \mu d}\right)}$$

*K* (m^2^/s) is the evaporation rate, which is typically calculated using the R-squared law (*R*_*d*_^2^ *– R*_0_^2^ *= Kt*),^40^ where *R*_*d*_ and *R*_0_ are the instantaneous and initial droplet radius, and *t* is the evaporation time. *D* (m^2^/s) is the diffusion coefficient of the nanoparticle within the droplet and *k_B_* and *T* are Boltzmann’s constant (1.38 × 10^–23^ J/K) and absolute temperature (295 K), respectively. Finally, μ is the initial droplet viscosity and d is the nanoparticle diameter.

When *Pe* ≫ 1, the rate of evaporation is significantly faster than the rate of nanoparticle diffusion, so there is slower redistribution of the nanoparticles within the droplet. Under such conditions, nanoparticles tend to accumulate near the liquid–air interface, leading to the formation of a shell either at or near the surface of the droplet.^41^ As the liquid continues to evaporate, the capillary pressure across the curved liquid interface may exceed the mechanical stability of the shell, resulting in buckling. However, if the capillary pressure is lower than the mechanical strength of the shell, then hollow spherical supraparticles may form. In contrast, when *Pe* is less than or equal to unity, the nanoparticles are distributed homogeneously during droplet drying, resulting in the formation of solid, dense, spherical supraparticles.^34,42–44^

A higher *Pe* associated with a higher degree of buckling can be obtained by increasing the evaporation rate (*Pe ∝ K*) at constant *D* or by reducing the nanoparticle diffusion coefficient $\left(Pe\propto \frac{1}{D}\right)$ at constant *K*, achieved by increasing the nanoparticle diameter $\left(\mathrm{since}\;D\propto \frac{1}{d}\right)$ .^20^ Miles et al.^39^ reported that less buckling occurs when using larger nanoparticles at constant *K,* which indicates that the degree of buckling increases with *D* under such conditions. Indeed, similar trends are observed for supraparticles formed when using binary mixtures of nanoparticles of differing size; the degree of buckling increases with *K* at fixed *D*_*avg*_ and increases with initial *D*_*avg*_ at fixed *K*.^20^ The *D*_*avg*_ can be calculated from the effective nanoparticle diameter (d_*p–eff*_) (see eq 2).^45^

2 $$D_{avg}=\frac{k_{B}T}{3\pi \mu d_{p-eff}}$$

and the thermal energy (*k_B_T*) and viscosity (μ).

For binary mixtures of nanoparticles and more complex mixtures, the d_*p–eff*_ can be calculated using the weighted harmonic mean relation for a mixture of nanoparticles of *n* sizes,^46^

$$d_{p-eff}=\overset{n}{\underset{i=1}{\sum }}\varphi_{i}{\left(\overset{n}{\underset{i=1}{\sum }}\frac{\varphi_{i}}{d_{i}}\right)}^{-1}$$ 3

Here φ is the nanoparticle concentration (% (w/w)), *n* is the number of distinct nanoparticle populations (i.e., *n* = 1, 2, 3, 4, and 5 for monomodal, bimodal, trimodal, tetramodal, and pentamodal size distributions, respectively) and d_*i*_ is the nanoparticle diameter for each population. An increase in the relative concentration of smaller nanoparticles compared to larger ones, while keeping the overall nanoparticle concentration constant, results in a higher mean diffusion coefficient (see eqs 2 and 3). This relationship, along with the discussion above, highlights a promising route for the controlled fabrication of supraparticles by modulating the relative concentration of nanoparticles of differing size within a polydisperse nanoparticle suspension without the need for any other additives such as surfactants or dissolved solutes. This approach offers a potentially decisive advantage over the surfactant-based strategies. More specifically, avoiding surfactants prevents contamination and reduces the risk of inadvertent colloidal destabilization.

In this paper, we use evaporation-induced self-assembly to prepare either spherical or buckled supraparticles from drying aqueous aerosol droplets containing sterically-stabilized diblock copolymer nanoparticles under controlled relative humidity (RH) at either 0% or 45%, where RH is varied to control the evaporation rate during droplet drying. Initially, we investigate the influence of the nanoparticle size distribution on the final supraparticle morphology by mixing samples of near-monodisperse nanoparticles of differing size at various relative concentrations to create well-defined bimodal, trimodal, tetramodal, and pentamodal size distributions. Such nanoparticle blends were carefully selected to maintain a constant mean diffusion coefficient for each mixture and evaporation was conducted under the same drying conditions. In particular, we show that the various supraparticle morphologies observed for a tetramodal size distribution are consistent with those previously reported for a bimodal size distribution, which supports our hypothesis that the diffusion coefficient calculated from the harmonic mean of the nanoparticle diameters can be used to predict the final morphology. However, we also identify limitations for this harmonic mean approximation when there is a relatively large difference in nanoparticle size. Under such circumstances, individual nanoparticle diffusion coefficients can dominate, thus leading to deviations from the expected supraparticle morphology based on the harmonic mean diffusion coefficient. To evaluate these effects, we analyze a series of nanoparticle mixtures comprising either tetramodal or bimodal size distributions.

## 2 Experimental Methods and Materials

The various experimental methods used for preparing the colloidal nanoparticles, fabricating and collecting supraparticles have been reported previously and are only briefly discussed here.

### 2.1 Materials

Dichloromethane, DMF, acetone and ethanol (ACS grade) were purchased from Fisher Scientific (UK) and used without further purification. All chemicals were used as received. Benzyl methacrylate (BzMA; 96%), 2-cyano-2-propyl dithiobenzoate (CPDB; >97%) and 4,4′-azobis­(4-cyanopentanoic acid) (ACVA; >98%) were purchased from Sigma-Aldrich (UK). Glycerol monomethacrylate (GMA, 99.5%) was kindly provided by GEO Specialty Chemicals (Hythe, UK) and was used as received. CD_3_OD (99.8%) and *d*_7_-DMF (99.8%) were purchased from Goss Scientific Instruments Ltd., UK. Deionized water (pH 6.8) was obtained using an Elga Elgastat Option 3A water purification system.

### 2.2 Synthesis of the PGMA Hydrophilic Precursor

This synthesis was based on a literature protocol.^47–51^ A round-bottomed flask was charged with GMA (30.0 g; 187 mmol), CPDB (0.829 g; 3.75 mmol; target degree of polymerization, DP = 60), ACVA (0.167 mg; 0.596 mmol; CPDB/ACVA molar ratio = 6.3), and ethanol (40.0 g), and then sealed using a rubber septum. This reaction vessel was purged with N_2_ gas for 30 min and placed in a preheated oil bath at 70 °C for 3 h,^52^ after which the polymerization was quenched by immersing the flask in an ice bath while exposing its contents to air. The resulting PGMA precursor (GMA conversion = 83%, *M*_n_ = 9,100 g mol^–1^, *M*_w_/*M*_n_ = 1.15, Figure S1) was purified by precipitation into excess dichloromethane to remove unreacted monomer and initiator residues. A mean degree of polymerization (DP) of 55 was calculated using ^1^H NMR spectroscopy (CD_3_OD) by comparing the integrated signals between 3.4 and 4.3 ppm assigned to the five pendant C–H protons of the GMA repeat units with that of the five aromatic protons between 7.4 and 8.0 ppm assigned to the dithiobenzoate end-group (Figure S1).

### 2.3 Synthesis of PGMA_55_–PBzMA_n_ Nanoparticles by RAFT Aqueous Emulsion Polymerization

A typical synthetic protocol for the preparation of PGMA_55_–PBzMA_1000_ nanoparticles at 10% w/w solids by RAFT aqueous emulsion polymerization is as follows. To a 6 mL glass vial containing a magnetic flea, PGMA_55_ precursor (50 mg, 5.26 μmol, 1 equiv), BzMA (926 mg, 5.26 mmol, 1000 equiv) and ACVA (0.4 mg, 1.31 μmol, 0.25 equiv) were added and dissolved in water (5.5 mL). The vial was sealed with a rubber septum and the reaction solution was purged with N_2_ gas for 30 min. The vial was then placed into an aluminum heating block that had been preheated to 70 °C. After 18 h, the polymerization was quenched by immersing the glass vial in liquid nitrogen and exposing its contents to air. An aliquot was removed for ^1^H NMR spectroscopy studies and SEC analysis using DMF eluent. DLS analysis was performed after dilution to an appropriate copolymer concentration. This synthetic protocol was repeated for [BzMA]/[PGMA_55_] molar ratios of 50, 100, 200, 400, 600, and 800 (Table 1). In each case, relatively high BzMA conversions (>98%) were confirmed by ^1^H NMR analysis, as indicated by the disappearance of the BzMA vinyl signals at 5.65–6.20 ppm (assigned NMR spectra are shown in the Supporting Information, see Figure S1).

**Table 1:** Summary of Characterization Data for a Series of PGMA–PBzMA Diblock Copolymer Nanoparticles Prepared via RAFT Aqueous Emulsion Polymerization of BzMA When Targeting 10% w/w Solids

Polymer composition	Target PBzMA DP	[Solids] (%w/w)	BzMA conv. (%)^a^	*M*_n,SEC_ (kDa)^b^	*M*_w_/*M*_n_ ^b^	*D*_h_ (nm)^c^	PD^c^
PGMA_55_ precursor	60	40	83	9.1	1.15	N/A	N/A
PGMA_55_–PBzMA_50_	50	10	>99	10.9	1.30	31 ± 2	0.19
PGMA_55_–PBzMA_100_	100	10	>99	13.1	1.36	42 ± 3	0.10
PGMA_55_–PBzMA_200_	200	10	>99	24.8	1.47	86 ± 3	0.05
PGMA_55_–PBzMA_400_	400	10	>99	46.4	1.42	122 ± 6	0.05
PGMA_55_–PBzMA_600_	600	10	>99	61.7	1.73	197 ± 8	0.07
PGMA_55_–PBzMA_800_	800	10	>99	78.8	1.48	215 ± 11	0.04
PGMA_55_–PBzMA_1000_	1000	10	>99	99.0	1.55	415 ± 24	0.18

a Determined by ^1^H NMR spectroscopy.

b Apparent number-average molecular weight, *M*_n_, and dispersity (*M*_w_/*M*_n_) calculated by SEC using PMMA standards and DMF eluent.

c *D*_h_ is the z-average hydrodynamic diameter and PD is the polydispersity index, as reported by DLS. [N.B. All copolymers are described in terms of their target diblock composition].

### 2.4 Characterization Techniques

#### 2.4.1 NMR Spectroscopy

^1^H NMR spectra were recorded in either CD_3_OD or *d*_7_-DMF at 400 MHz using a Bruker Ascend 400 spectrometer. Proton chemical shifts are reported as δ in parts per million (ppm) and are expressed relative to tetramethylsilane (TMS) at δ = 0 ppm.

#### 2.4.2 Size Exclusion Chromatography (SEC)

Analysis was performed using an Agilent 1260 Infinity GPC system equipped with an Agilent guard column (PLgel 5 μm) and two Agilent Mixed-C columns (PLgel 5 μm). Detection involved either a differential refractive index (RI) detector or a UV–visible detector (set to λ = 305 nm). The mobile phase was DMF (HPLC-grade) containing 10 mM LiBr at 60 °C at a flow rate of 1.0 mL min^–1^. Number-average molecular weights (*M*_n_), weight-average molecular weights (*M*_w_) and dispersities (*Đ* = *M*_w_/*M*_n_) were calculated using a series of near-monodisperse poly­(methyl methacrylate) calibration standards ranging from 800 to 2,200 000 Da.

#### 2.4.3 Dynamic Light Scattering (DLS)

Measurements were performed using a Malvern Zetasizer Nano ZS instrument equipped with a 4 mW He–Ne 633 nm laser and an avalanche photodiode detector. Backscattered light was detected at an angle of 173° and data were recorded at a copolymer concentration of 1% w/w at 25 °C. Malvern Zetasizer software v7.11 was used to calculate z-average hydrodynamic diameters (*D*_h_) via the Stokes–Einstein equation, which assumes perfectly monodisperse, noninteracting spherical particles. Data were averaged over at least three consecutive runs with at least ten measurements being recorded for each run.

Dynamic light scattering (DLS) studies confirmed unimodal size distributions, with z-average hydrodynamic diameters ranging from 31 to 415 nm on increasing the target PBzMA DP from 50 to 1000 (Figure 1b, Table 1). SEC analysis indicated a high chain extension efficiency for the PGMA precursor (Figure 1c) and a monotonic increase in diblock copolymer *M*_n_ was observed when targeting higher PBzMA DPs (see Table 1).

**Figure 1:**
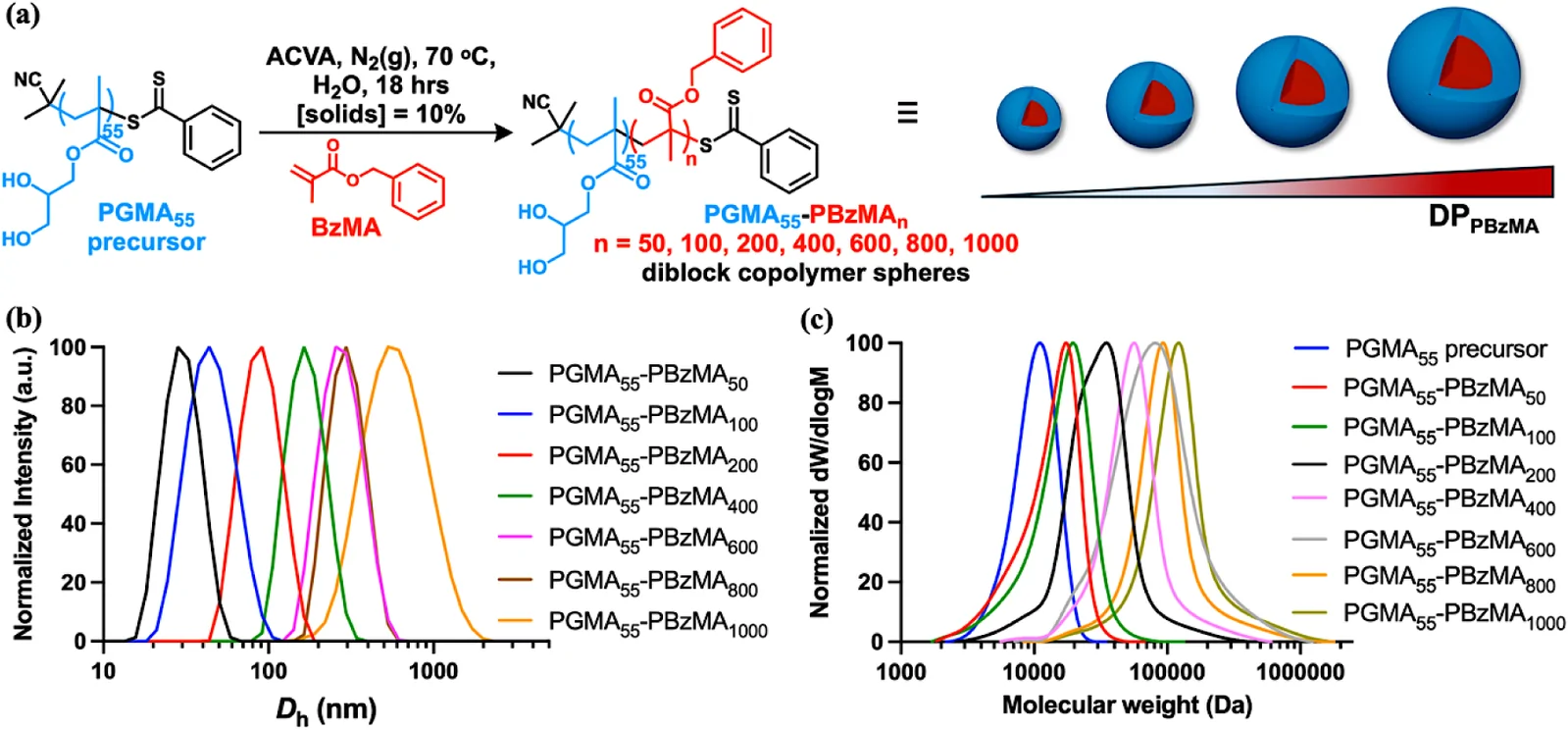
Synthesis and characterization of a series of poly­(glycerol monomethacrylate)-poly­(benzyl methacrylate) (PGMA–PBzMA) diblock copolymer nanoparticles of varying size via RAFT aqueous emulsion polymerization. (a) Schematic representation of the preparation of PGMA–PBzMA_n_ nanoparticles (where *n* = 50, 100, 200, 400, 600, 800 or 1000). The z-average nanoparticle diameter increases monotonically on increasing the target DP for the core-forming PBzMA block. Abbreviations: ACVA = 4,4′-azobis­(4-cyanovaleric acid), BzMA = benzyl methacrylate. (b) Intensity-weighted particle size distributions determined by DLS for PGMA–PBzMA_n_ nanoparticles (*n* = 50, 100, 200, 400, 600, 800 or 1000) and (c) SEC curves (refractive index detector) recorded for the corresponding diblock copolymer chains dissolved in DMF. An SEC curve obtained for the PGMA_55_ precursor is included as a reference.

### 2.5 Preparation of Supraparticles via Falling Droplet Column (FDC)

Supraparticles were prepared and collected using a Falling Droplet Column (FDC) at RH 0% and 45%. Previous studies have reported that the evaporation kinetics measured using FDC are in good agreement with those obtained from comparative kinetic electrodynamic balance (CK-EDB).^52^ Thus, only a brief discussion of the FDC experimental setup is provided here.

The FDC consists of a 50 cm vertical transparent glass column with an inner cross-sectional area of 4 cm^2^, a green light laser (λ = 532 nm), a droplet-on-demand (DoD) dispenser, and a microscopic glass slide at the bottom for collecting the dried supraparticles. A chain of uniform aqueous aerosol droplets loaded with a series of mixtures of various nanoparticles at a given overall concentration were dispensed using the same DoD for each mixture. The chain of droplets, initial diameter ∼38 ± 2 μm, was introduced from the top of the column and illuminated vertically by the laser beam, which is used to guide and align the droplet path within the center of the column. As droplets evaporate while falling through the column, supraparticles are formed and then collected on a microscopic glass slide positioned at the base of the column. A regulated flow of nitrogen gas (comprising a mixture of dry and moist nitrogen) was passed through the column to maintain the required drying conditions (RH = 0 or 45%). An RH probe was installed inside the column to monitor the actual RH in real time. The temperature was measured using a K-type thermocouple and was maintained at 22 °C for all experiments.

The morphology of the dried supraparticles was assessed by SEM. The glass slides containing supraparticles were sputter-coated with a thin layer of silver. Then SEM analysis was performed using a JEOL, JSM-IT300 instrument operating under ultrahigh vacuum using an electron beam voltage of 5–15 kV, a beam current of 5–20 pA, and a working distance of 10–11 mm.

It is assumed that the drying droplets maintain their initial spherical morphology when falling under gravity. This is supported by the Bond number (Bo) of ∼10^–4^ calculated for the present study. This Bo value is well below unity, which is consistent with the condition required to maintain a spherical morphology.^53^

## 3 Results and Discussion

### 3.1 Preparation of Supraparticles Using Complex Mixtures of Nanoparticles of Varying Size

First, supraparticles were prepared by drying droplets (∼38 μm initial diameter) containing near-monodisperse nanoparticles of 122 nm diameter at a nanoparticle concentration of 2.0% w/w for RH = 0% or 45%. As discussed earlier, the aim of the present study is to produce supraparticle morphologies using various nanoparticle mixtures while maintaining the same harmonic mean diameter. To achieve an equivalent harmonic mean diameter from a bimodal mixture, we used nanoparticles with mean diameters of 86 and 197 nm at the same nanoparticle concentration of 1.0% w/w, for which we calculate a harmonic mean diameter of 120 ± 6 nm. To prepare the corresponding trimodal, tetramodal and pentamodal nanoparticle mixtures, we selected nanoparticles of appropriate size and adjusted their relative concentration accordingly, while keeping the overall nanoparticle concentration constant at 2.0% w/w (Table S1). The specific nanoparticle diameter and nanoparticle concentration required for each mixture are shown in the first column of Figure 2. The second and third column presents SEM images recorded for dried supraparticles formed at an RH of either 0% or 45%. Regardless of the type of nanoparticle mixture (i.e., whether monomodal, bimodal, trimodal, tetramodal or pentamodal), Figure 2 indicates that the supraparticle morphology remains unchanged for a given set of drying conditions. This supports our hypothesis that the harmonic mean diameter calculated for complex nanoparticle mixtures is a useful parameter for predicting the final morphology.

**Figure 2:**
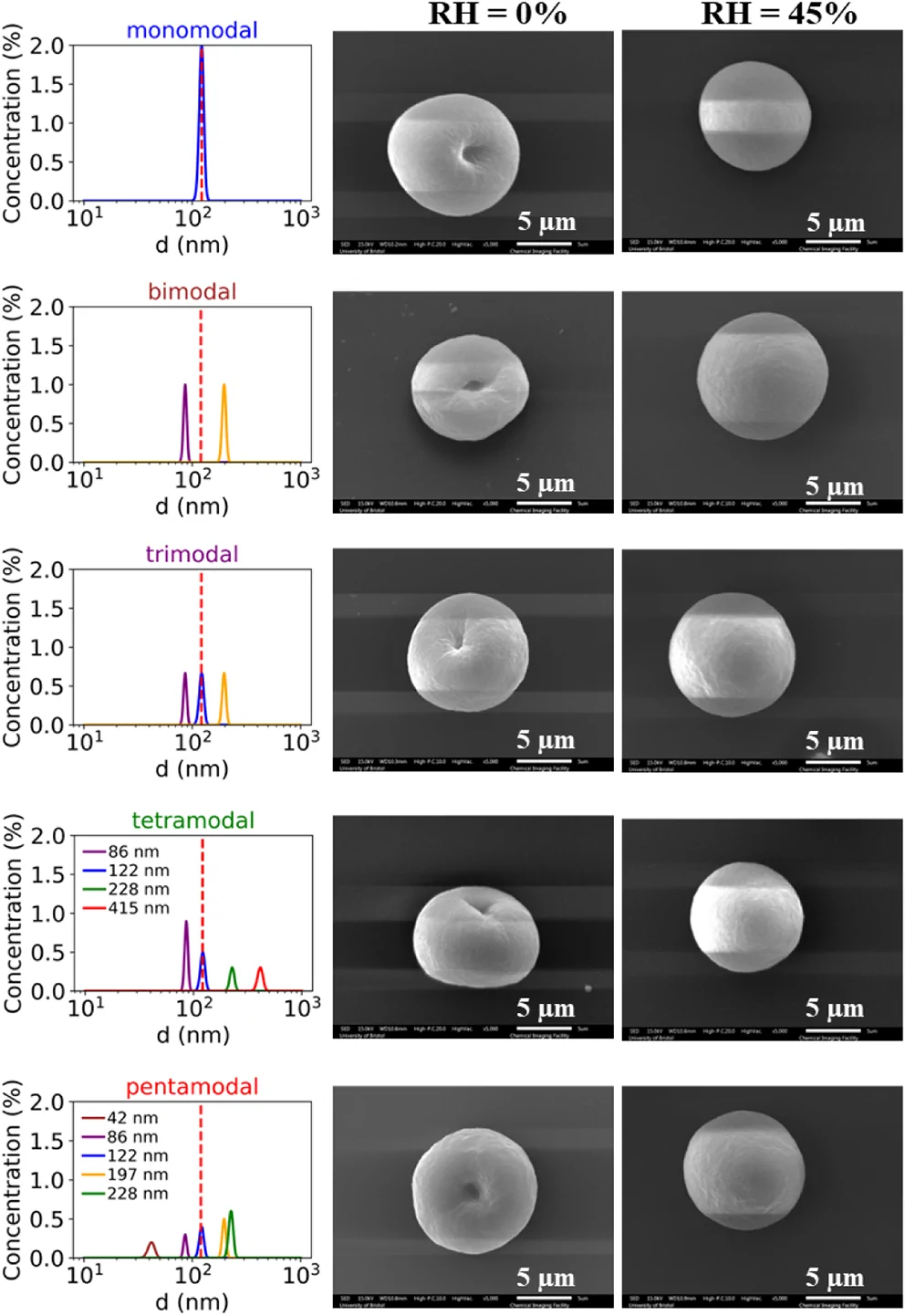
Preparation of well-defined supraparticles using multicomponent mixtures of nanoparticles. Representative SEM images are recorded for RH = 0% and RH = 45% when using monomodal, bimodal, trimodal, tetramodal, or pentamodal nanoparticle size distributions, respectively. The vertical red dotted line indicates the effective mean diameter for each respective mixture.

To further quantify the morphology obtained from such nanoparticle mixtures, we analyzed the circularity of the supraparticles. The circularity is defined as the ratio of the area of the maximum included circle to the area of the minimum enclosing circle and it provides a robust parameter to assess any deviation from perfect sphericity.^20^ The circularity is determined from SEM analysis, which requires that the supraparticles have a consistent orientation on deposition, i.e., a clear top view. However, since supraparticles are collected under free-fall conditions, they do not always deposit in a consistent orientation. Therefore, when estimating the mean circularity under specific drying conditions, we include only those supraparticles with consistent orientations. For buckled supraparticles, this corresponds to selecting particles with circularity values below 0.40; values above this threshold are attributed to supraparticles with inconsistent orientations, as indicated by the gray bars shown in Figure 3. To ensure consistency, the same selection criteria were applied across all samples. While this necessarily involves manual selection, it was performed systematically to minimize bias by selecting supraparticles with similar top- view orientations. Typically, 50–150 supraparticles were analyzed for each condition to ensure statistical reliability. The distribution of circularity for each modal system is presented in Figure S2, together with the corresponding mean values and standard deviations, while the mean values across all modal systems are shown in Figure 3. The mean circularities calculated for supraparticles prepared when drying at 0% RH using monomodal, bimodal, trimodal, tetramodal and pentamodal nanoparticle populations are 0.25 ± 0.04, 0.21 ± 0.05, 0.25 ± 0.03, 0.29 ± 0.03, 0.24 ± 0.06 respectively, (Figure S2). In striking contrast, the corresponding circularities are 0.89 ± 0.02, 0.91 ± 0.03, 0.89 ± 0.03, 0.89 ± 0.02, 0.91 ± 0.03 when the same droplet drying experiments were conducted at 45% RH (Figure S2). The mean circularity calculated for all complex nanoparticle mixtures is 0.23 ± 0.05 at 0% RH and 0.90 ± 0.03 at 45% RH. The corresponding probability density plots suggest that the supraparticles exhibit highly consistent morphologies (Figure S3). Although circularity offers a convenient metric for comparing morphologies, it is inherently limited to 2D projections and does not fully resolve 3D features such as shell thickness and internal structure.

**Figure 3:**
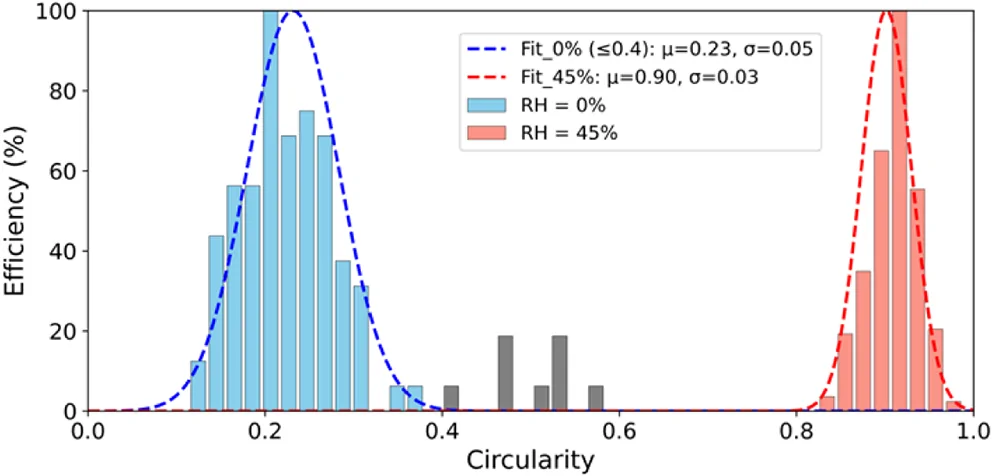
Histogram and normal distributions for circularity data determined for each complex mixture of nanoparticles. The blue and red dashed lines represent the normal distribution fit for RH = 0% and RH = 45%, respectively. The μ and σ denote the mean and standard deviation, respectively. The circularity for each nanoparticle system is provided in the Supporting Information.

To summarize, the supraparticle morphologies remain consistent for the various complex nanoparticle mixtures, provided that the effective harmonic mean diameter remains constant at 120 ± 6 nm. The low circularities obtained at 0% RH indicate the formation of non-spherical (or buckled) supraparticles owing to the higher capillary pressure during the relatively fast rate of evaporation. In contrast, the high circularities observed at 45% RH reflect hollow spherical supraparticles, which are formed at slower rates of evaporation when Pe > 0. These observations are consistent with the higher degree of buckling anticipated for higher *K* values (for a given nanoparticle diffusion coefficient).

The remarkably similar circularities observed for a given RH value suggests that both shell formation and the drying dynamics are primarily governed by the environmental conditions and effective nanoparticle diffusivity, rather than the precise nature of the multimodal nanoparticle size distribution. Hence the supraparticle morphology can be predicted by *tuning the environmental conditions* and the *mean nanoparticle diffusion coefficient* alone. The prediction of morphology when systematically varying the latter parameter is discussed in the next section.

### 3.2 Effect of Systematic Variation of the Mean Nanoparticle Diffusion Coefficient on the Supraparticle Morphology

In principle, the mean diffusion coefficient, *D_avg_*, for a complex mixture of nanoparticles of varying size is inversely proportional to the effective nanoparticle diameter, d_*p–eff*_. Thus, we sought to systematically vary the effective nanoparticle diameter to understand how the mean nanoparticle diffusivity affects the final supraparticle morphology. More specifically, we selected three distinct mixtures of nanoparticles, with each mixture composed of four different types of nanoparticles present at the same 0.5% w/w concentration (see Figure 4, first column). Hence, the overall nanoparticle concentration was again fixed at 2.0% w/w. Depending on the z-average diameter for each individual nanoparticle population, the effective nanoparticle diameters calculated for these three mixtures are 52 nm, 82 nm, and 137 nm (Figure 4 and Table S2).

**Figure 4:**
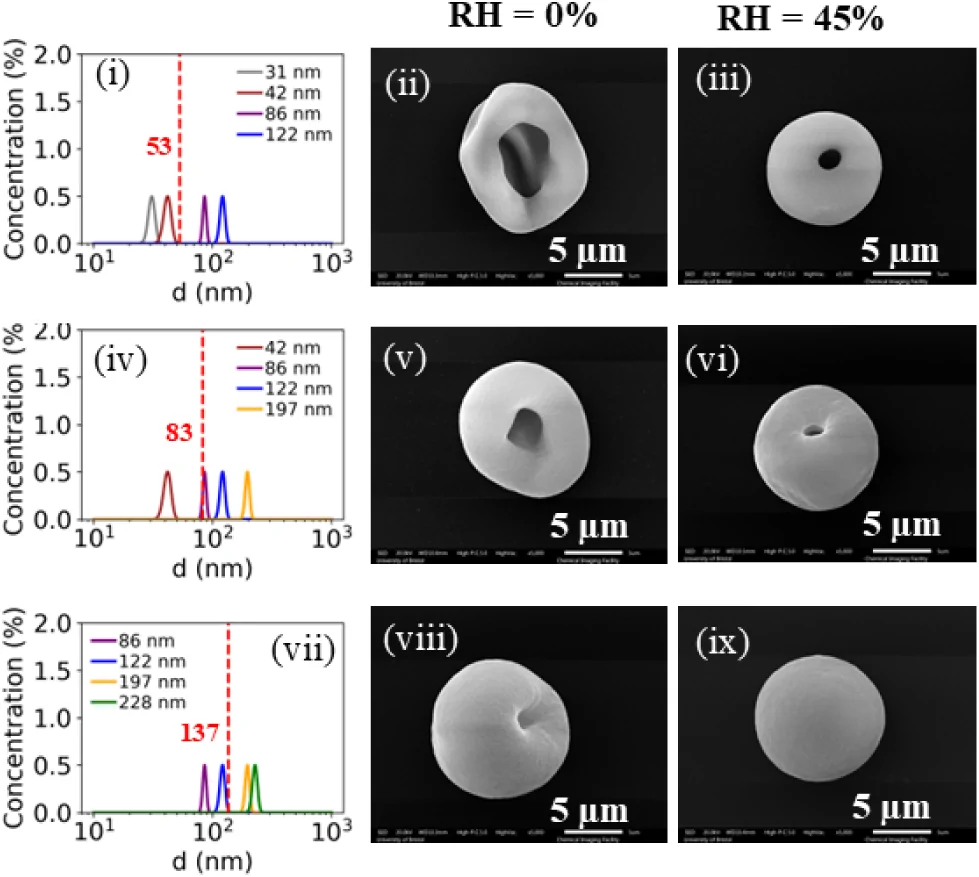
Representative SEM images recorded for supraparticles prepared using nanoparticles comprising a tetramodal size distribution made up of nanoparticles of differing size but identical relative and overall nanoparticle concentrations. The red numbers indicate the effective mean diameter of these nanoparticles in each case.

Typical corresponding supraparticle morphologies observed after drying at either 0% RH or 45% RH are shown in the second and third columns of Figure 4. The supraparticle morphologies vary from highly buckled to either less buckled or spherical as the effective nanoparticle diameter is increased, regardless of the drying conditions. This trend is consistent with our prior study of supraparticle formation using a monomodal population of near-monodisperse nanoparticles.^39^ At constant RH, increasing the nanoparticle diameter (and thus reducing the mean diffusion coefficient) leads to higher nanoparticle propensity at the surface of the drying droplets and the resulting relatively thick shell reduces the degree of buckling. For a slower rate of evaporation (which is inversely proportional to RH), reduced buckling is observed for a given effective nanoparticle diameter.

To assess such changes in morphology, we calculated the circularity of the dried supraparticles. Circularities obtained for the three tetramodal nanoparticle mixtures are indicated on the map of circularity with varying initial mean diffusion coefficient and evaporation rate in Figure 5 adapted from Mahato et al.^20^ The results (see black circles in Figure 5) are in excellent agreement with this prior study, which was validated for binary mixtures of nanoparticles of differing size.^20^ Thus, this extends the fidelity of the predictive framework to more complex mixtures of nanoparticles that exhibit well-defined multimodal size distributions. In summary, the supraparticle morphology can be controlled by adjusting two key parameters: the mean nanoparticle diffusion coefficient (as calculated from the effective nanoparticle diameter) and the rate of evaporation (which depends on the RH and temperature). Although the harmonic mean framework captures the overall trends in supraparticle morphology, it does not explicitly account for differences in packing structure that may arise from the nanoparticle size distribution. For example, multimodal systems may exhibit higher packing efficiency or local structural heterogeneity compared to monomodal systems. Such effects are likely to influence internal density and mechanical properties. However, this aspect is not the aim of the present study and would require further structural characterization. Exploring these aspects in more detail should be a fruitful direction for future work.

**Figure 5:**
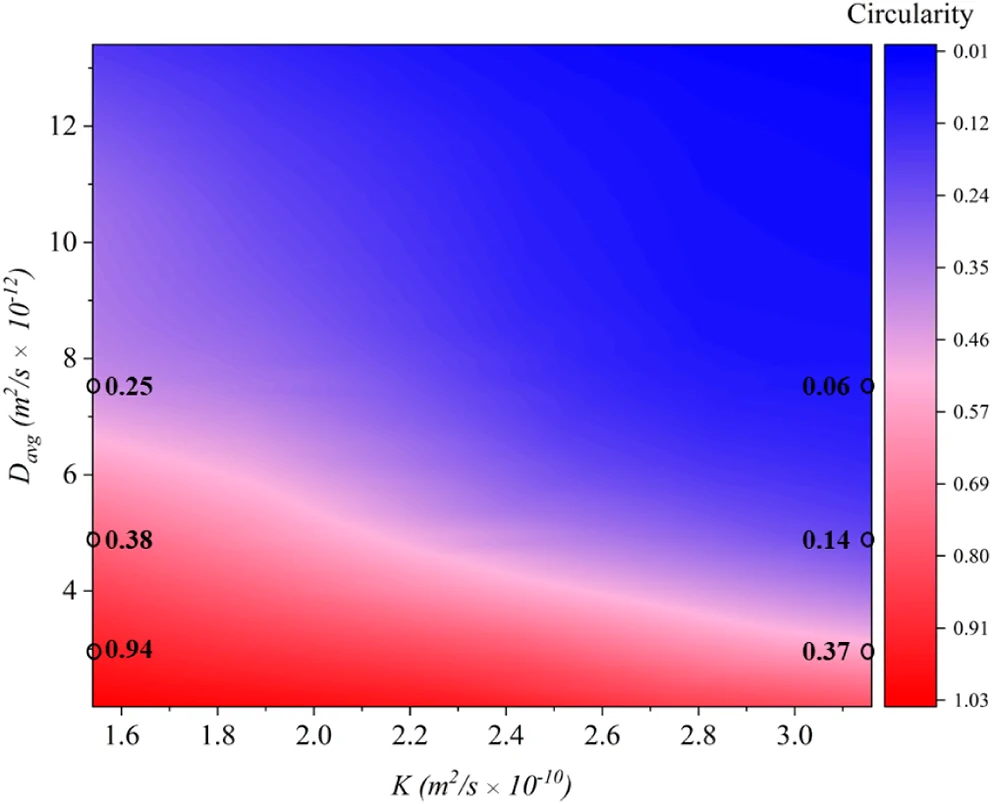
Variation in circularity adapted from Mahato et al. Journal of Colloid and Interface Science, 2025, 682, 251–262, used under CC BY 4.0.^20^ The black circles represent circularities recorded at 0% (*K* ∼ 3.16 × 10^–10^ m^2^/s) and 45% RH (*K* ∼ 1.54 × 10^–10^ m^2^/s) when using a complex mixture of nanoparticles with a tetramodal size distribution and an effective nanoparticle diameter of 53 nm (*D_avg_* ∼ 7.7 × 10^–12^ m^2^/s), 83 nm (*D_avg_* ∼ 4.9 × 10^–12^ m^2^/s) and 137 nm (*D_avg_* ∼ 3.0 × 10^–12^ m^2^/s). The values shown represent the corresponding circularities under each condition.

### 3.3 Testing the Limits of Morphology Prediction Using the Mean Diffusion Coefficient Approach

In the previous sections we have demonstrated the preparation of well-defined supraparticles from aqueous droplets containing complex mixtures of nanoparticles and shown how the final morphology can be predicted by controlling the mean nanoparticle diffusion coefficient and the rate of evaporation. However, it is not yet clear whether this approach is broadly applicable.

To investigate the scope and limits of our predictive approach, we selected two nanoparticle mixtures each comprising a tetramodal size distribution and an effective diameter of approximately 120 nm. The first mixture (denoted as 86-tetramodal) included nanoparticles with z-average diameters of 86 nm, 121 nm, 228 nm, and 415 nm present at concentrations of 0.9%, 0.5%, 0.3%, and 0.3% w/w, respectively (Figure 6, first column). For the second tetramodal sample (denoted as 31-tetramodal), the 86 nm nanoparticles are replaced with 31 nm nanoparticles and the nanoparticle concentrations are accordingly adjusted to 0.3%, 0.2%, 0.7%, and 0.8% w/w, respectively (Table S3) to maintain the same effective nanoparticle diameter (Figure 6, second column).

**Figure 6:**
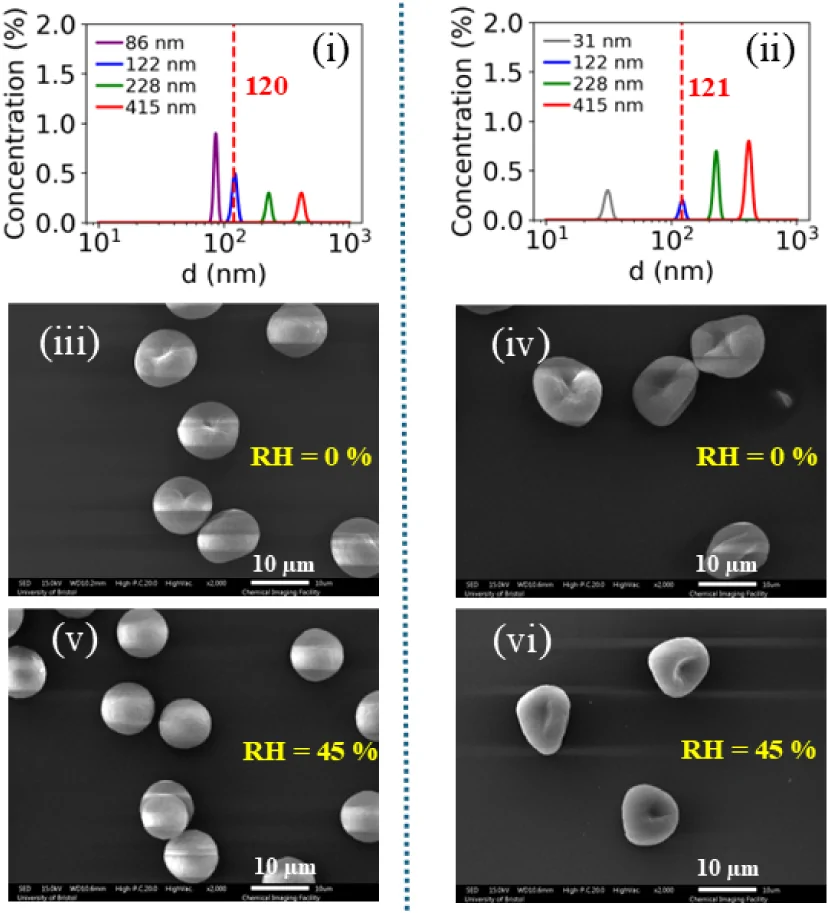
Comparison of the supraparticle morphologies obtained when using the same effective diameter for a complex mixture of nanoparticles comprising a tetramodal size distribution different size and concentration. Concentration and morphologies observed for supraparticles dried at RH = 0% and RH = 45% when using 86-tetramodal (i, iii, v) and 31-tetramodal (ii, iv, vi) complex mixtures.

Despite employing the same effective nanoparticle diameter, the resulting supraparticles clearly exhibit different morphologies. At 0% RH, supraparticles obtained from the 31- tetramodal mixture are highly buckled (Figure 6iv), while supraparticles derived from the 86-tetramodal mixture have a discernibly lower degree of buckling (Figure 6iii); this is consistent with the predicted morphology according to the harmonic mean diameter model. Similarly, drying the 86-tetramodal mixture at 45% RH yields the predicted spherical supraparticles (Figure 6v). However, the 31-tetramodal mixture unexpectedly produces buckled supraparticles (Figure 6vi). This discrepancy is attributed to the extreme size difference between the smallest and largest nanoparticles in the latter mixture. Notably, the diffusion coefficient of the 31 nm nanoparticles is nearly 2 orders of magnitude greater than that of the 415 nm nanoparticles (Figure S4). As a result, differential diffusion leads to spatial segregation during evaporation: the smaller nanoparticles diffuse faster and hence accumulate more at the droplet surface, forming a relatively thin shell which leads to buckling. The more slowly diffusing larger particles remain uniformly distributed throughout the droplet volume. It is important to note that supraparticle morphology is influenced by several parameters, including interparticle interactions, size ratio, relative nanoparticle concentration, and the Péclet number.^26,31,34^ In the present study, sterically-stabilized diblock copolymer nanoparticles were used to minimize attractive interparticle interactions, thus allowing the effects of diffusion and the rate of evaporation to be isolated. Under such conditions, the observed behavior is primarily governed by differences in nanoparticle diffusivity at a given evaporation rate. However, for large size disparities, the marked diffusivity contrast between nanoparticle populations promotes preferential surface segregation of the smaller nanoparticles. In principle, the extent of such segregation should depend on the nanoparticle size ratio, relative number concentration, and drying conditions, which collectively determine shell formation and the final supraparticle morphology. To further rationalize this phenomenon, the number of each type of nanoparticles within the droplet is calculated (see the Supporting Information and Table S3, column 5 for further details). For the 86-tetramodal mixture, the number of 86 nm nanoparticles is 100× of magnitude greater than the number of 415 nm nanoparticles. However, for the 31-tetramodal mixture, there are more than a 1000× of the smallest nanoparticles for each of the largest nanoparticles. Hence both the size and number concentration of the nanoparticles affects the supraparticle morphology.

To provide more insight into the impact of size disparity on supraparticle morphology, we investigated systematically how the relative numbers of small and large nanoparticles can radically change the supraparticle shape using only two types of nanoparticles (31 nm and 415 nm). The overall nanoparticle concentration was held constant at 2.0% w/w and the relative concentration of the larger nanoparticles was systematically reduced from 2.0% to 0% (Table S4). The resulting supraparticle morphologies are shown in Figure 7, with magnified surface regions highlighted in yellow. Regardless of the drying conditions, the 31 nm nanoparticles always produced highly buckled supraparticles while the 415 nm nanoparticles invariably produced spherical supraparticles. When the 415 nm nanoparticles comprise ≥1.9% (w/w) of the mixture (i.e., when there are 132 small nanoparticles or fewer for each large nanoparticle), the resulting supraparticles remain spherical. However, as the relative concentration of larger nanoparticles is lowered below this threshold, the degree of buckling gradually increases. Notable deviations in buckling are observed when comparing such supraparticles to those prepared using nanoparticles with the same harmonic mean diameter but either monomodal or multimodal size distributions. This suggests that extreme differences in nanoparticle diameter promotes size-dependent interfacial segregation and hence a higher degree of buckling. Indeed, high-magnification SEM images (see inset images in Figure 7) recorded for such supraparticles indicates that the surface concentration of larger nanoparticles quickly becomes negligible as the number concentration of smaller nanoparticles is increased. However, larger nanoparticles remain visible on the surface in the central regions of the supraparticles (e.g., see the inset image for the mixture containing 31 nm and 415 nm nanoparticles at concentrations of 1% and 1% w/w, respectively), while they are not readily visible in the outer regions. These observations are consistent with preferential accumulation of the smaller nanoparticles at or near the droplet interface during drying. A more quantitative assessment of the surface composition would require additional characterization techniques and is unfortunately beyond the scope of the present study. The high surface concentration of the smaller nanoparticles is consistent with the shell-thinning mechanism that facilitates greater buckling.

**Figure 7:**
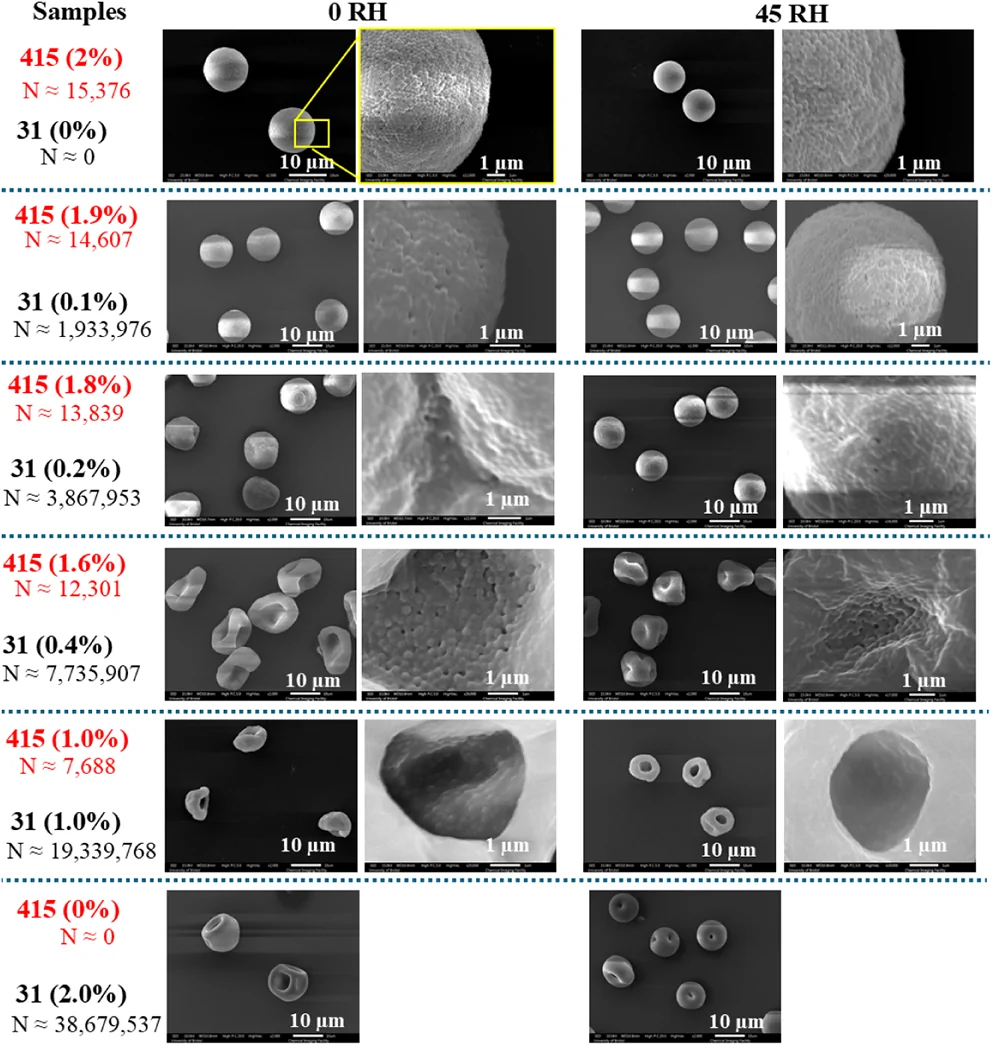
Representative SEM images recorded for supraparticles prepared by evaporation of aqueous aerosol droplets containing various binary mixtures of 415 nm and 31 nm nanoparticles at a fixed overall nanoparticle concentration of 2.0% w/w. The first column shows the compositions and corresponding number of nanoparticles, for example, 415 (1.9%) N ≈ 14,607 denotes 415 nm nanoparticles at a concentration of 1.9% w/w, which corresponds to 14,607 nanoparticles per droplet. The second and third columns represent the supraparticles that are formed at 0% and 45% RH respectively, with the corresponding magnified regions highlighted in yellow.

Overall, this final set of experiments suggests that both size disparity and relative number concentration can play a crucial role in determining the supraparticle morphology. More specifically, the harmonic mean approximation fails when the diffusion coefficients of two (or more) types of nanoparticles differ by more than an order of magnitude owing to preferential surface segregation of the smallest nanoparticles. The interpretation is limited to indirect evidence rather than direct compositional mapping, based on SEM analysis of morphology plus systematic trends in nanoparticle diffusion coefficients and the relative nanoparticle number concentration. In such cases, accurate predictions of the final supraparticle morphology must not only account for the overall system properties but also for local compositional differences.

## 4 Conclusions

We report a robust strategy for controlling the formation of supraparticles with defined morphologies by manipulating the nanoparticle size distribution and drying conditions during evaporation-induced self-assembly. In particular, supraparticles with the same spherical or buckled morphology can be reproducibly prepared by employing complex nanoparticle mixtures exhibiting multimodal size distributions with a constant effective diameter (i.e., identical mean diffusion coefficient) and relative humidity (i.e., evaporation rate). We estimate the effective nanoparticle diameter using the harmonic mean approximation, which enables the final supraparticle morphology to be predicted for moderate size differences between the various nanoparticle populations. However, for systems with relatively large size differences, this approach fails to predict the observed morphology. In such cases, diffusion of the smallest nanoparticles predominates, leading to diffusion-driven segregation according to size. This is particularly evident when the diffusion coefficients of the smallest and largest nanoparticles differ by more than 2 orders of magnitude. The smaller particles preferentially accumulate near the droplet interface, resulting in thinner shells and a higher degree of buckling. We also show that both nanoparticle size differences and the relative number concentration influence the final supraparticle morphology. When large nanoparticles are present at a sufficiently high concentration, they thicken the shell and hence reduce buckling. Conversely, if their concentration is too low, the more rapidly diffusing small nanoparticles dominate, leading to a high degree of buckling. While the harmonic mean framework works well for the sterically-stabilized nanoparticles studied here, its applicability to other systems may be limited. For example, stronger interparticle interactions, aggregation, or gelation in inorganic or charged colloids could influence particle mobility and shell formation during drying, leading to deviations from the predicted behavior. Nevertheless, when such effects are negligible, the harmonic mean approximation provides an appropriate first-order approach from which deviations can be assessed.

Overall, these findings suggest that the final supraparticle morphology can be fine-tuned simply by controlling the nanoparticle size distribution and the drying conditions. This new approach eliminates the need for surfactants or other additives, enabling the clean and scalable production of structured micron-sized supraparticles for potential applications such as drug delivery, photonics, sensing, and catalysis.

## Supplementary Material



## Notes

Tables for different polydisperse systems and their corresponding values, table for tetramodal mixture with increasing effective diameter, table for variation in concentration in binary mixtures, ^1^H NMR spectra, circularity for each polydisperse mixture, and justification for choosing the harmonic mean approximation (PDF)
