# Supplementary material for: Control over the Morphology of Dried Supraparticles from the Evaporation-Induced Self-Assembly of Aerosol Droplets Containing Polydisperse Nanoparticles
Source: Langmuir. 2026 Aug 29;42(36):26227–36. doi: 10.1021/acs.langmuir.6c02636 (PMC13585123; doi:10.1021/acs.langmuir.6c02636)
Supplement: Supplementary file 1 [file la6c02636_si_001.pdf]

Supporting Information

Control over the morphology of dried supraparticles from the evaporation-induced self-assembly of aerosol droplets containing polydisperse nanoparticles

Lukesh K. Mahato[1], Panagiotis G. Georgiou[2], Barnaby E. A. Miles[1], Sorrel K. Haughton[1], Jake G. Edmans [2], Rachael E. H. Miles[1], Steven P. Armes[2 *] & Jonathan P. Reid[1 *]*

[1]School of Chemistry, University of Bristol, Bristol, BS8 1TS, UK
[2]School of Mathematical and Physical Sciences, Dainton Building, University of Sheffield, Sheffield, S3 7HF, UK

Corresponding author's email: lukesh.mahato@bristol.ac.uk (L. K. Mahato), s.p.armes@sheffield.ac.uk (S. P. Armes) and j.p.reid@bristol.ac.uk (J. P. Reid)

Contents:

(1) Table for different polydisperse system and their corresponding value (Table S1)

(2) Table for tetramodal mixture with increasing effective diameter (Table S2) and for same effective diameter (Table S3)

(3) Table for variation in concentration in binary mixtures for limitation test (Table S4)

(4) ^{1}H NMR spectra (Figure S1)

(5) Circularity and it's probability density for each polydisperse mixture (Figure S2 and S3)

(6) Diffusion coefficients with nanoparticles size

(7) Calculation of number of nanoparticles

(8) Justification for choosing the harmonic mean approximation

Table S1. Different polydisperse system and their corresponding values. The first column represents the number of distinct nanoparticles used in the mixture. The fourth, fifth and sixth columns denotes the respective individual nanoparticle concentrations, the overall nanoparticle concentration and the effective harmonic mean diameter calculated for each mixture, respectively.

Number of monodisperse nanoparticles	Diblock copolymer composition	d (nm)	φ_i (% w/w)	φ_t (% w/w)	$d_{p\text{-}eff}$ (nm)
1	$PGMA_{55}$-$PBzMA_{400}$	122 ± 6	2.0	2	122 ± 6
2	$PGMA_{55}$-$PBzMA_{200}$	86 ± 3	1.0	2	120 ± 5
	$PGMA_{55}$-$PBzMA_{600}$	197 ± 8	1.0		
3	$PGMA_{55}$-$PBzMA_{200}$	86 ± 3	0.667	2	120 ± 6
	$PGMA_{55}$-$PBzMA_{400}$	122 ± 6	0.667		
	$PGMA_{55}$-$PBzMA_{600}$	197 ± 8	0.667		
4	$PGMA_{55}$-$PBzMA_{200}$	86 ± 3	0.9	2	120 ± 8
	$PGMA_{55}$-$PBzMA_{400}$	122 ± 6	0.5		
	$PGMA_{55}$-$PBzMA_{800}$	228 ± 11	0.3		
	$PGMA_{55}$-$PBzMA_{1000}$	415 ± 24	0.3		
5	$PGMA_{55}$-$PBzMA_{100}$	42 ± 3	0.2	2	120 ± 5
	$PGMA_{55}$-$PBzMA_{200}$	86 ± 3	0.3		
	$PGMA_{55}$-$PBzMA_{400}$	122 ± 6	0.4		
	$PGMA_{55}$-$PBzMA_{600}$	197 ± 8	0.5		
	$PGMA_{55}$-$PBzMA_{800}$	228 ± 11	0.6		

Table S2. Variation of the effective diameter for each tetramodal mixture of nanoparticles.

Number of monodisperse nanoparticles	Diblock copolymer composition	d (nm)	φ_i (% w/w)	φ_t (% w/w)	$d_{p\text{-}eff}$ (nm)
4	$PGMA_{55}$-$PBzMA_{50}$	31 ± 2	0.5	2	53 ± 3
	$PGMA_{55}$-$PBzMA_{100}$	42 ± 3	0.5		
	$PGMA_{55}$-$PBzMA_{200}$	86 ± 3	0.5		
	$PGMA_{55}$-$PBzMA_{400}$	122 ± 6	0.5		
4	$PGMA_{55}$-$PBzMA_{100}$	42 ± 3	0.5	2	86 ± 5
	$PGMA_{55}$-$PBzMA_{200}$	86 ± 3	0.5		
	$PGMA_{55}$-$PBzMA_{400}$	122 ± 6	0.5		
	$PGMA_{55}$-$PBzMA_{600}$	197 ± 8	0.5		
4	$PGMA_{55}$-$PBzMA_{200}$	86 ± 3	0.5	2	137 ± 7
	$PGMA_{55}$-$PBzMA_{400}$	122 ± 6	0.5		
	$PGMA_{55}$-$PBzMA_{600}$	197 ± 8	0.5		
	$PGMA_{55}$-$PBzMA_{800}$	228 ± 11	0.5		

Table S3. Variation of different nanoparticles within various tetramodal mixtures with the same effective diameter. The representations are the same as those indicated in Table S1.

Number of monodisperse nanoparticles	Diblock copolymer composition	d (nm)	φ_i (% w/w)	Number of nanoparticles per droplet	φ_t (% w/w)	$d_{p\text{-}eff}$ (nm)
4	$PGMA_{55}$-$PBzMA_{200}$	86 ± 3	0.9	773,720	2	120
	$PGMA_{55}$-$PBzMA_{400}$	122 ± 6	0.5	152,964		
	$PGMA_{55}$-$PBzMA_{800}$	228 ± 11	0.3	13,925		
	$PGMA_{55}$-$PBzMA_{1000}$	415 ± 24	0.3	2,306		
4	$PGMA_{55}$-$PBzMA_{50}$	31 ± 2	0.3	5,801,930	2	121
	$PGMA_{55}$-$PBzMA_{400}$	122 ± 6	0.2	61,185		
	$PGMA_{55}$-$PBzMA_{800}$	228 ± 11	0.7	32,492		
	$PGMA_{55}$-$PBzMA_{1000}$	415 ± 24	0.8	6,150		

Table S4: Variation of nanoparticle concentration within binary mixtures comprising 31 nm and 415 nm nanoparticles. The representations are the same as those given in Table S1.

Number of monodisperse nanoparticles	Diblock copolymer composition	*d* (nm)	φ_i (% w/w)	φ_t (% w/w)	$d_{p\text{-}eff}$ (nm)
1	$PGMA_{55}$-$PBzMA_{1000}$	415 ± 24	2.0	2	415
2	$PGMA_{55}$-$PBzMA_{50}$	31 ± 2	0.05	2	315
	$PGMA_{55}$-$PBzMA_{1000}$	415 ± 24	1.95		
2	$PGMA_{55}$-$PBzMA_{50}$	31 ± 2	0.1	2	255
	$PGMA_{55}$-$PBzMA_{1000}$	415 ± 24	1.9		
2	$PGMA_{55}$-$PBzMA_{50}$	31 ± 2	0.2	2	184
	$PGMA_{55}$-$PBzMA_{1000}$	415 ± 24	1.8		
2	$PGMA_{55}$-$PBzMA_{50}$	31 ± 2	0.3	2	144
	$PGMA_{55}$-$PBzMA_{1000}$	415 ± 24	1.7		
2	$PGMA_{55}$-$PBzMA_{50}$	31 ± 2	0.4	2	118
	$PGMA_{55}$-$PBzMA_{1000}$	415 ± 24	1.6		
2	$PGMA_{55}$-$PBzMA_{50}$	31 ± 2	1	2	57
	$PGMA_{55}$-$PBzMA_{1000}$	415 ± 24	1		
1	$PGMA_{55}$-$PBzMA_{50}$	31 ± 2	2	2	31

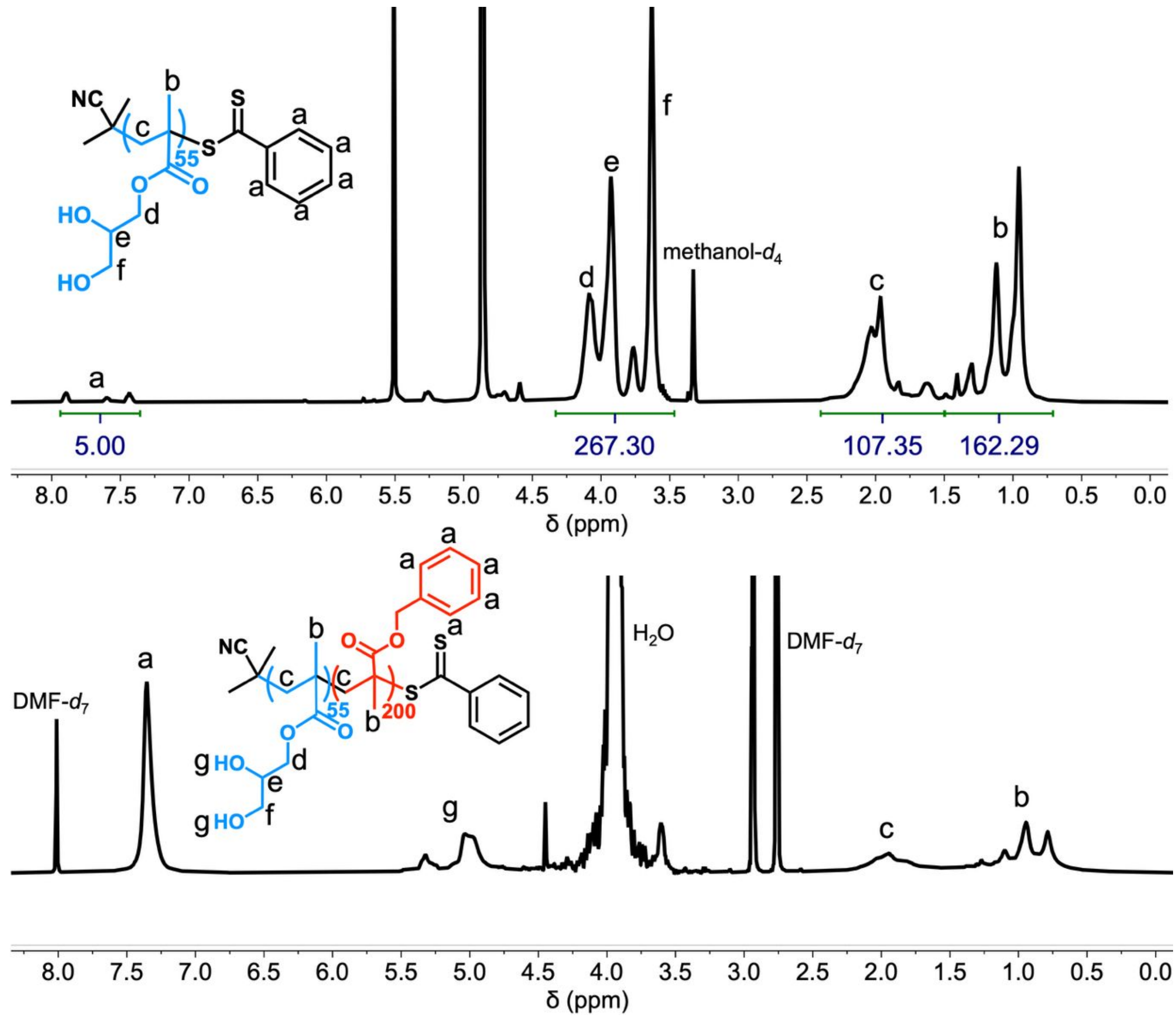

Figure S1. ^{1}H NMR spectra recorded for the $PGMA_{55}$ precursor (in CD_3OD) and the unpurified $PGMA_{55}$-$PBzMA_{200}$ diblock copolymer (in d_7-DMF).

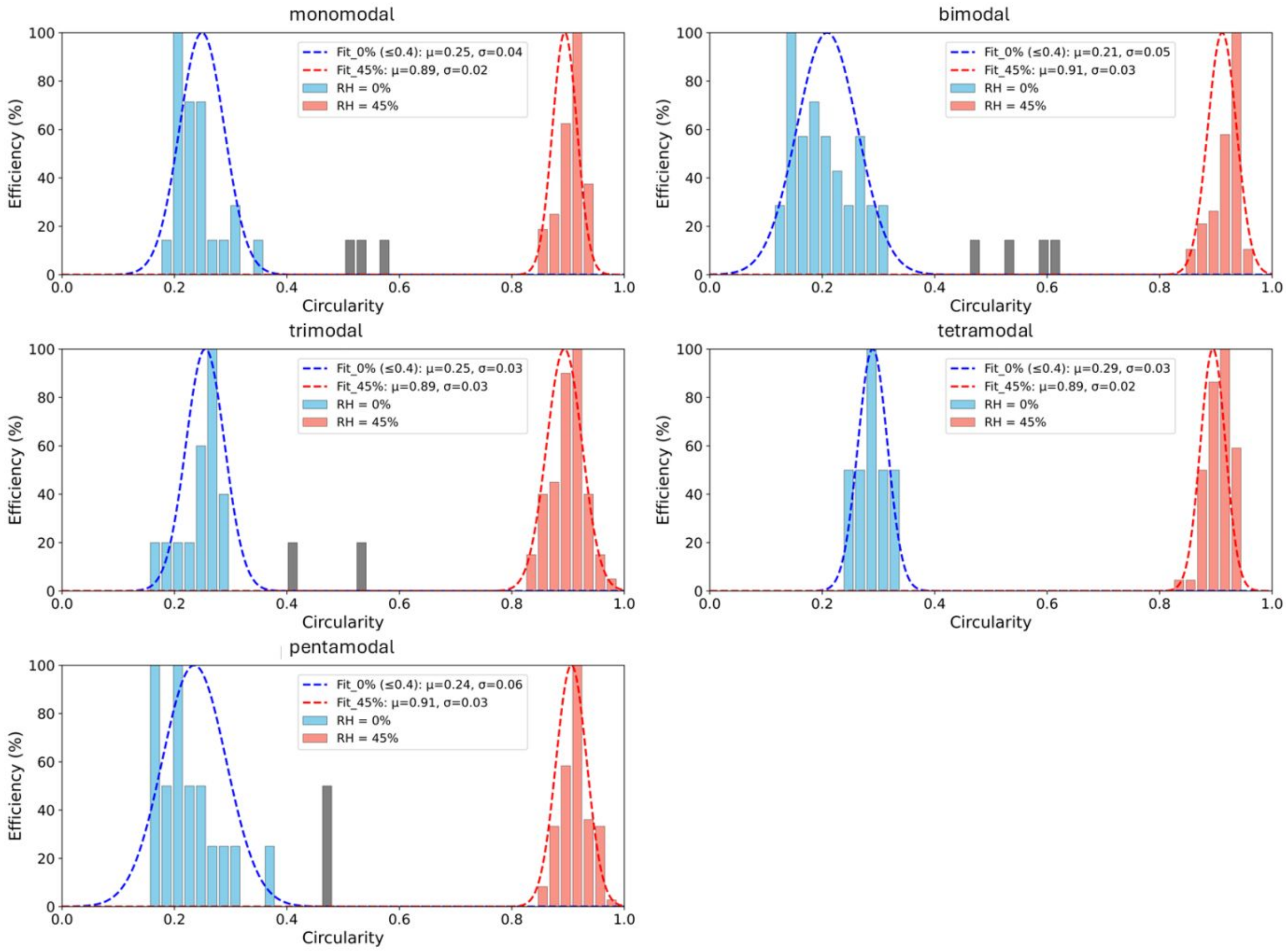

Figure S2. Circularity calculated for each monomodal, bimodal, trimodal, tetramodal and pentamodal mixture of nanoparticles at 0% and 45% RH.

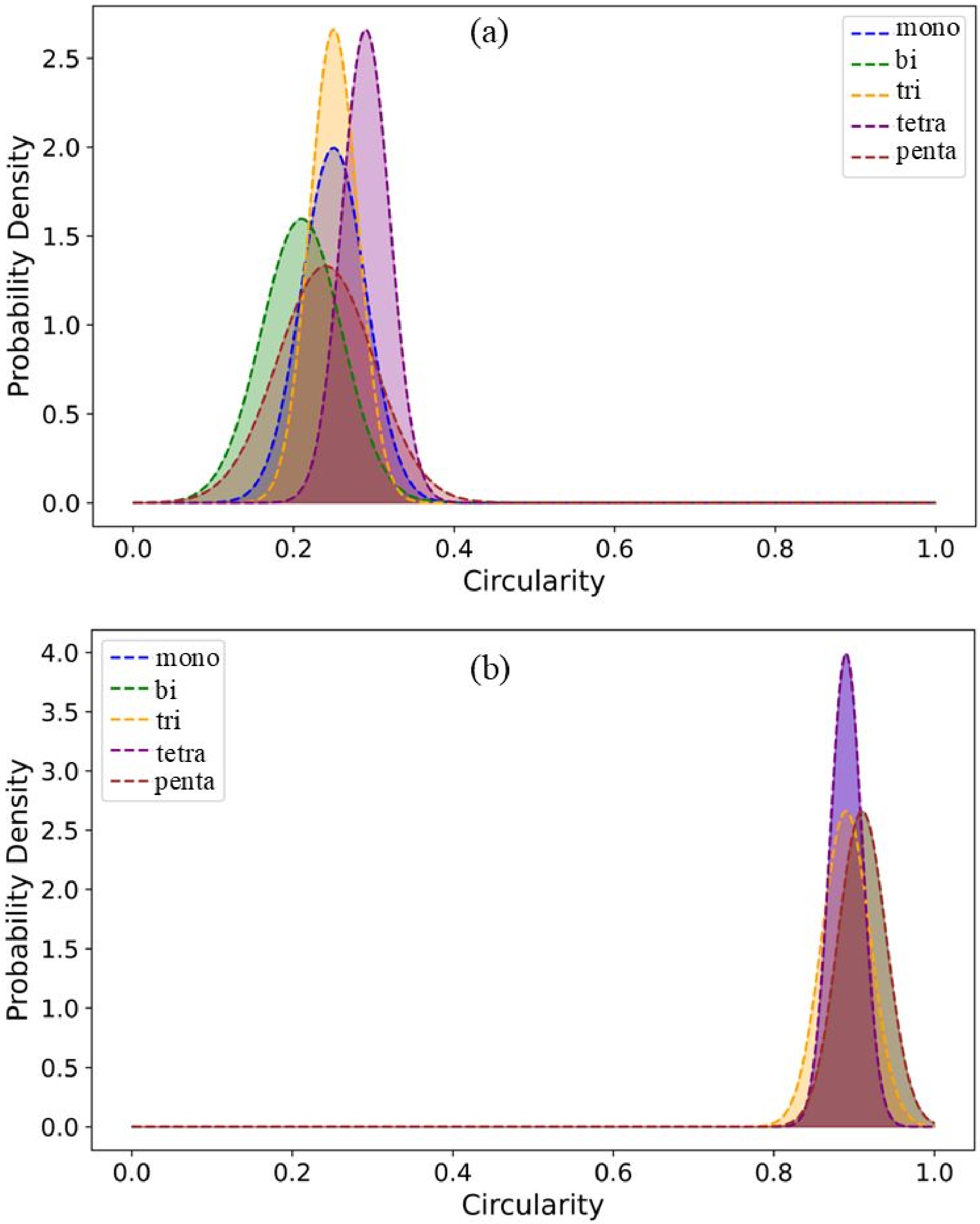

Figure S3. Distribution of circularity for all supraparticles prepared using monomodal, bimodal, trimodal, tetramodal and pentamodal mixture of nanoparticles at (a) 0% and (b) 45% RH.

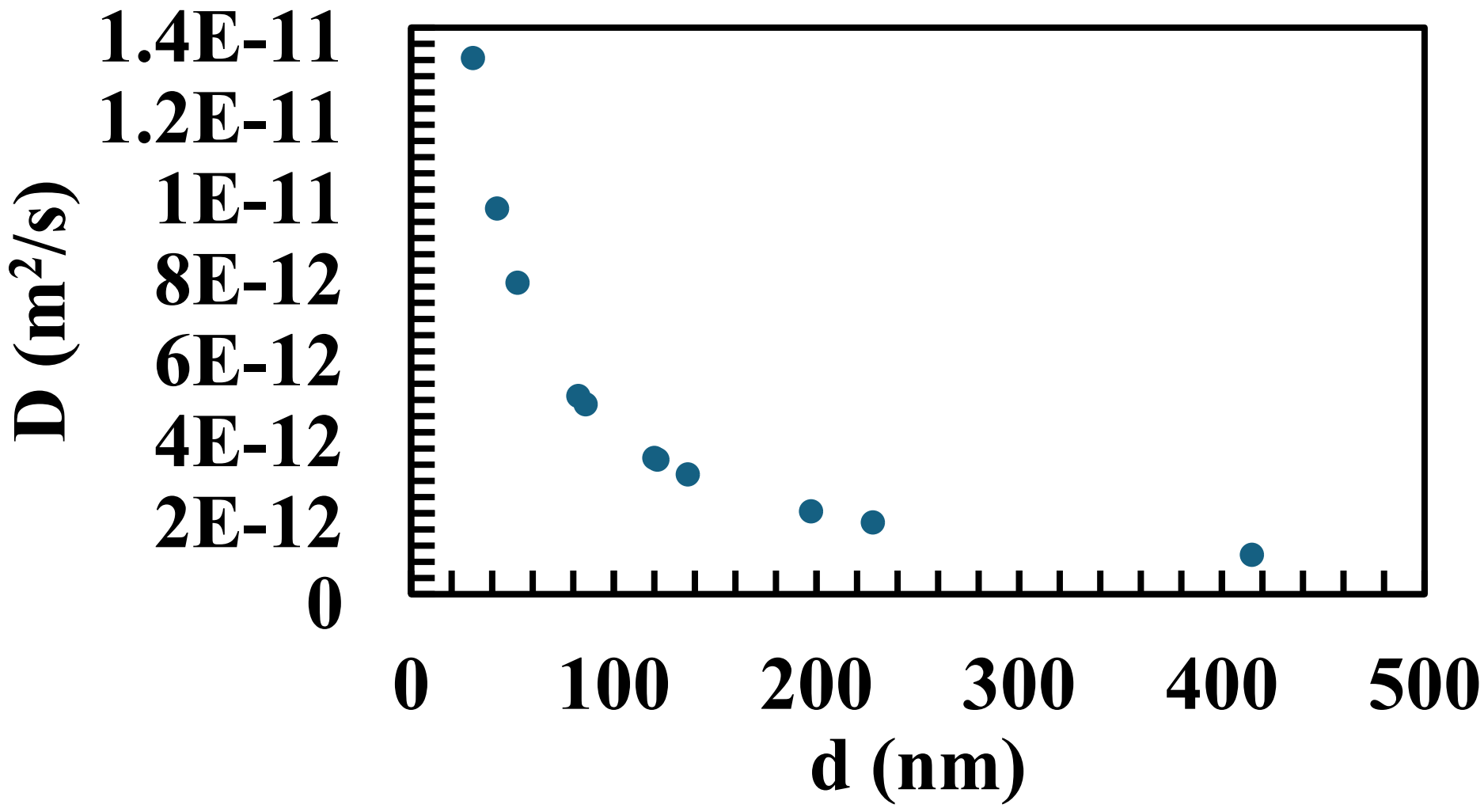

Figure S4. Variation of diffusion coefficients with nanoparticle diameter.

Calculation for number of nanoparticles

The number of nanoparticles is the ratio of the volume of solids in a single droplet to the volume of an individual nanoparticle. The volume of solids in a droplet can be calculated by simply multiplying the volume fraction and volume of droplet using this equation:

$$N = \frac{V_{\text{solids in droplet}}}{V_{\text{p}}} = \frac{\phi\, V_{\text{droplet}}}{V_p}$$

where

$V_{\text{droplet}} = \frac{4}{3}\pi\left(\frac{d_{\text{droplet}}}{2}\right)^3$ is the droplet volume, d_{droplet} is the droplet diameter (~ 38 μm), ϕ is the nanoparticle volume fraction within the droplet, and $V_p$is the volume of a single nanoparticle:

$$V_p = \frac{4}{3}\pi\left(\frac{d_p}{2}\right)^3 = \frac{\pi}{6}\, d_p^3$$

Here, d_p is the mean nanoparticle diameter.

Hence it follows that

$$N \approx \frac{\phi\, V_{\text{droplet}}}{\frac{\pi}{6}\, d_{\text{mean}}^3}$$

Justification for choosing the harmonic mean approximation

Curtiss and Bird (1999) proposed an equation for calculating the mean diffusion coefficient for a multicomponent mixture of liquid droplets in terms of the molar fraction. Similarly, for a polydisperse mixture of colloidal particles comprising *n* number of nanoparticles of differing size and concentration ($\varphi_1, \varphi_2, \ldots\ldots \varphi_n$), the mean diffusion coefficient (D_{avg}) can be calculated using Eq. (S1):

$$D_{avg} = \frac{\varphi_1}{\varphi} D_1 + \frac{\varphi_2}{\varphi} D_2 + \ldots\ldots + \frac{\varphi_n}{\varphi} D_n \tag{S1}$$

Here φ is the total concentration ($\varphi = \varphi_1 + \varphi_2 + \ldots + \varphi_n$) of particles in solution, D_1, D_2, and D_n are the diffusion coefficients of the respective nanoparticles ($D_1 = \frac{K_b T}{3\pi \mu d_1}$), ($D_2 =$

$\frac{K_bT}{3\pi\mu d_2}$), and ($D_n = \frac{K_bT}{3\pi\mu d_n}$), , and d_1, d_2, and d_n are the mean diameters of the 1, 2 and n nanoparticles. Substituting values for D_1, D_2 and D_n into Eq. (S2);

$$D_{avg} = \frac{\varphi_1}{\varphi}\frac{k_BT}{3\pi\mu d_1} + \frac{\varphi_2}{\varphi}\frac{k_BT}{3\pi\mu d_2} + \ldots\ldots + \frac{\varphi_n}{\varphi}\frac{k_BT}{3\pi\mu d_n}$$

(S2)

$$D_{avg} = \frac{k_BT}{3\pi\mu}\left\{\frac{1}{\varphi}\left(\frac{\varphi_1}{d_1} + \frac{\varphi_2}{d_2} + \ldots\ldots\frac{\varphi_n}{d_n}\right)\right\}$$

(S3)

$$D_{avg} = \frac{k_BT}{3\pi\mu d_{p-eff}}$$

(S4)

Where d_{p-eff} is the effective diameter for the polydisperse nanoparticles, as calculated using Eq. (S5).

$$\frac{1}{d_{p-eff}} = \left\{\frac{1}{\varphi}\left(\frac{\varphi_1}{d_1} + \frac{\varphi_2}{d_2} + \ldots\ldots\frac{\varphi_n}{d_n}\right)\right\}$$

(S5)

$$d_{p-eff} = \sum_{i=1}^{n}\varphi_i\left(\sum_{i=1}^{n}\frac{\varphi_i}{d_{pi}}\right)^{-1}$$

(S6)

Eq. (S6) is the same as the weighted harmonic mean equation.

Curtiss C. F., Bird R. B.. Diffusion-stress relations in polymer mixtures. *J. Chem. Phys.* **1999**, 111, 10362-10370. doi:10.1063/1.480385
